# Prevalence and Determinants of Gastro‐Esophageal Reflux Disease Among Medical Students: A Cross‐Sectional Study From Pakistan

**DOI:** 10.1002/hsr2.73459

**Published:** 2026-10-31

**Authors:** Easha Masood, Sana Fatima, Rumman Ashraf, Muddassir Khalid, Fatima Tul‐Zahra, Mishaal Zarar, Sumia Fatima, Fred Segawa

**Affiliations:** ^1^ Foundation University Medical College Islamabad Pakistan; ^2^ Rawalpindi Medical University Rawalpindi Pakistan; ^3^ Nishtar Medical University Multan Pakistan; ^4^ Makerere University College of Health Sciences Kampala Central Region Uganda

**Keywords:** epidemiology (Q000453), esophagitis (D004941), gastric acid (D005744), medical (D013337), students, the databases include gastro‐esophageal reflux (D005764)

## Abstract

**Background and Aims:**

Gastro‐esophageal reflux disease (GERD) significantly impacts the quality of life and has shown increasing prevalence globally. Despite its increasing burden, limited data is available on medical students who are at higher risk due to lifestyle habits such as irregular eating patterns and stress. This study aimed to assess the association between lifestyle factors, perceived stress and GERD.

**Methods:**

A cross‐sectional study was conducted with a sample size of 242 (CI: 95%; ERR: 5%), using the Cochran formula. Convenience sampling was used, and a structured questionnaire was distributed in November 2024 to inquire about demographics, risk factors, probable GERD (GERDQ), and the Perceived Stress Scale (PSS‐10). Participants were categorized as follows: GERD: cutoff ≥ 8; stress: low (0–13), moderate (14–26), high (27–40). Multivariate regression analysis was performed to identify the predictors of GERD, with reporting of odds ratio and 95% confidence interval.

**Result:**

Out of the 249 responses received, 212(85.1%) were female, and 189(75.9%) had a healthy BMI (18–25 kg/m2). The calculated GERD prevalence was 20.5% (n = 51), whereas 31 (12.4%) participants reported high levels of perceived stress. In bivariate analysis, GERD and midnight snacking (*p* = 0.007), mode of exercise (*p* = 0.034), and perceived stress (*p* = 0.025) were significant. In the multivariate analysis, only midnight snacking (AOR = 7.62, 95% CI: 1.60–36.21) and mode of exercise (AOR = 3.997, 95% CI: 1.53–10.46) remained statistically significant.

**Conclusion:**

Midnight snacking and exercise patterns are significant predictors of GERD. These findings suggest that lifestyle behavior contributes to the disease symptoms, and future longitudinal studies may benefit from evaluating the effectiveness of interventions targeting these factors, such as stress management programs/support groups, lifestyle modifications and disease‐related awareness.

## 1 Introduction

Gastro‐esophageal reflux disease is the backward flow of stomach acid into the esophagus. In the absence of appropriate treatment, it can lead to serious problems such as esophageal mucosal injury and Barrett's metaplasia, significantly impacting the patient's quality of life. [1, 2] The main cause of this condition is weakening of the defences relative to the body's innate protective mechanism, e.g., laxity of the lower esophageal sphincter (LES), and the key risk factors that expedite the onset of the disease include obesity, lack of exercise, smoking, and consumption of caffeinated or carbonated drinks. Mealtime irregularities, intermittent and untimely snacking, and eating large amounts of fatty food in one sitting can contribute to its development [3, 4].

GERD with pyrosis (heartburn) and regurgitation being the most common. [5] As it is challenging to properly and reliably detect GERD in patients, much of the diagnosis depends on patient history and investigations like upper GI endoscopy [6]. Changing a person's lifestyle to eliminate all the things that exacerbate their condition is the primary and most important part of not only the treatment but also prevention [7]

Globally, GERD is a common condition that affects a significant proportion of the population, with a worldwide prevalence of 13.98%. However, the burden of GERD varies substantially across different regions and populations, with the Western world experiencing higher rates (19.55% in North America and 14.12% in Europe) than Asia (12.92%) [8]. Interestingly, the prevalence of GERD in Pakistan (which lies in Asia) ranges from 22.2% to 24.0%, according to some hospital studies, but the data is limited. [9]

A bibliometric analysis of the trends in GERD research reveals that 8,964 articles have been published, with the USA leading the production at 35.74%. In Asia, the leading countries include China (9.00%), Japan (8.94%), and South Korea (4.24%). [10]

Medical students seem to be susceptible to GERD because of various environmental and psychological factors, like sedentary lifestyle due to long studying hours, stressful exam periods, sleep deprivation, and irregular mealtimes, which affect their daily life activities, such as sleep, oral intake, and work, leading to impaired quality of life and diminished productivity. [11] They often neglect to seek professional help for GERD and dyspeptic symptoms, either downplaying their severity or self‐medicating, which may worsen their condition [12].

In Pakistan's context, these factors may have a unique implication when considering the intense academic pressure and limited mental health support present. Previous studies have mainly been hospital‐based and may not reflect the burden of GERD among younger, otherwise healthy individuals. Therefore, our research aimed to estimate the prevalence of probable GERD among medical students in a public university of Pakistan and to explore its association with demographics, lifestyle behaviors, and perceived stress.

## 2 Methodology

### 2.1 Study Design and Setting

This study employed a quantitative, descriptive cross‐sectional design to assess the prevalence of GERD among undergraduate medical students of Pakistan. This research was conducted over 6 months (October 2024‐ March 2025) to ensure a comprehensive analysis within a defined timeframe. The cross‐sectional nature of the study allowed for the collection of data at a single point in time, providing a snapshot of GERD prevalence within the target population. Additionally, this study was planned and reported in accordance with the STROBE checklist (Strengthening the Reporting of Observational Studies in Epidemiology) [13].

### 2.2 Sample Size

The sample size was calculated based on a previous study that reported a 17.1% prevalence of GERD in a similar population [13]. Using this estimate, a 95% confidence level, and a 5% error margin, the minimum sample size calculated using the Cochran formula was 220 students. Considering a no‐response rate of 10%, the final sample size came out to be 242. A total of 249 completed questionnaires were received and analyzed in this study. The calculated sample size was deemed sufficient for accurate prevalence estimation with the specified precision; however, the non‐probability sampling reduces the representativeness and external validity of the results.

### 2.3 Data Collection Tools and Procedure

The questionnaire consisted of four sections: (a) demographic data such as sex, academic year of study, and BMI; and (b) a diagnostic tool for GERD, which is the GERD questionnaire (GERDQ) [14]. The fourth section focuses on the lifestyle and risk factors that contribute to the development of GERD. Finally, Cohen's Perceived Stress Scale (PSS) was included in Section (d) [15]. Incomplete questionnaires were excluded from the study.

The GERDQ is a validated and widely used questionnaire for screening probable GERD in epidemiological studies. It consists of six questions: four positive questions to measure GERD symptoms (heartburn, regurgitation, sleep disturbance caused by heartburn or regurgitation, and use of over‐the‐counter medications); and two negative questions (epigastric pain and nausea). It uses a four‐graded Likert scale (0–3) to score the occurrence of four positive interpreters and a reversed Likert scale (3‐0) for two negative interpreters of GERD, giving a total GERDQ score range of 0–18. The calculation of GERD's influence also includes sleep disturbance and OTC medication use, resulting in a separate ‘impact score’ which ranges from 0 to 6. [16] This questionnaire had a sensitivity of 65% and specificity of 71%. [17] The scoring procedure was re‐checked for consistency, with Cronbach's α of 0.785, showing acceptable internal consistency.

The following section includes questions regarding risk factors such as obesity, physical activity, smoking, and consumption of caffeinated or carbonated drinks, adapted from a study carried out in Egypt in 2023 [13].

Similarly, the Perceived Stress Scale (PSS) is a validated stress assessment tool, standardly used in student and healthcare populations. Established in 1983, this tool continues to be a popular choice for understanding how various situations can trigger emotional turmoil and perceived stress. This scale asks questions about emotions and thoughts over the last month. Individual score range of PSS was determined as follows: a score of 0 to 13 was considered less stress, a score from 14 to 26 was considered moderate stress, and a score from 27 to 40 was considered high perceived stress. [18] A good internal consistency was demonstrated by PSS (Cronbach's α = 0.836).

Subject experts reviewed the questionnaire for contextual appropriateness for Pakistani medical students, and no modifications were deemed necessary. Using a broad inclusion criterion, reaching a wide range of participants using various online platforms (institutional channels and student networks, e.g. WhatsApp and Facebook groups) helped diversify geographical representation and reduce selection bias to an extent. Before proceeding to the main questionnaire, eligibility screening questions were asked. Individuals with pre‐existing health conditions such as diagnosed gastrointestinal disease (e.g., peptic ulcer disease, inflammatory bowel disease, gastric cancer), chronic medical illnesses such as ischemic heart disease, chronic kidney or liver disease etc, any previous gastrointestinal surgery, pregnancy, or taking medications like NSAIDs were instructed not to proceed with the survey and were excluded. Employing two previously validated questionnaires and an adapted questionnaire from a preceding study, as mentioned above, helped minimize measurement bias. Likewise, anonymous responses encouraged the participants to answer each question truthfully.

A non‐random convenience sampling technique was used to select the participants. To increase diversity, the survey link was disseminated across multiple academic and student networking groups, and students from both public and private medical colleges in Pakistan were approached. However, due to the overlapping nature of these platforms, the exact number of students invited cannot be accurately estimated. Furthermore, the questionnaire was administered via Google Form with all fields marked mandatory to avoid incomplete responses. The settings were configured to allow a single response per participant, preventing duplicate entries.

### 2.4 Statistical Analysis

SPSS (version 27.0) was used for the data analysis [19]. Descriptive statistics, such as percentages, frequencies, means, and standard deviations, were used to summarize the demographic data, perceived stress levels, and other risk factors. Categorical variables are reported as frequency and proportion (n [%]), and group comparisons were performed using the chi‐square test. Normality of continuous variables was assessed using the Shapiro–Wilk test. GERDQ and PSS scores were not normally distributed; thus, Spearman's rank correlation was used. To investigate the relationship between different variables and the presence of GERD symptoms, Pearson's chi‐square test was employed. All statistical tests were two‐sided, with a significance threshold set at *p* < 0.05. Variables were added to the multivariable logistic regression model if *p* < 0.2 in bivariate analysis, and adjusted odds ratios with confidence intervals were reported.

### 2.5 Ethical Consideration

Ethical approval was obtained from the Ethical Review Board (ERB) of Rawalpindi Medical University on 5th November, 2024 (Reference No. PH‐46‐45). Informed consent was obtained from each individual, and participation was voluntary. The study included only those who consented to participate in the study. Anonymity and confidentiality were maintained throughout the process. The questionnaire did not include questions that required personal or identifying information.

## 3 Result

A total of 249 responses were analyzed in this study, of which females comprised 85.1% (n = 212) and males 14.9% (n = 37). The highest participation was observed in the 2nd‐year MBBS (36.5%), followed by the 3rd‐year (32.9%). Of all respondents, 75.9% (n = 189) were healthy (BMI: 18‐25), whereas 10.0% were overweight. The distribution of GERD status across demographic characteristics (gender, year of study, BMI) and lifestyle factors (dietary habits, physical exercise, smoking and stress level) are also shown in Table 1. Nearly two‐thirds of participants −71.5% (n = 178) had moderate stress, with 16.1% and 12.4% having low and high perceived stress, respectively.

**Table 1:** Association of baseline characteristics and perceived stress among study participants with GERD status.

Characteristics	Category	Total n (%)	GERD‐negative n (%)	GERD‐positive n (%)	*p* value
**Gender**	Female	212 (85.1)	167 (78.8)	45 (21.2)	0.486
	Male	37 (14.9)	31 (83.8)	6 (16.2)	
**Year of study**	1st Year MBBS	34 (13.7)	23 (67.6)	11 (32.4)	0.402
	2nd Year MBBS	91 (36.5)	74 (81.3)	17 (18.7)	
	3rd Year MBBS	82 (32.9)	68 (82.9)	14 (17.1)	
	4th Year MBBS	22 (8.8)	18 (81.8)	4 (18.2)	
	5th Year MBBS	20 (8.0)	15 (75.0)	5 (25.0)	
**BMI category**	Underweight (< 18)	32 (12.9)	26 (81.3)	6 (18.8)	0.795
	Healthy (18–25)	189 (75.9)	150 (79.4)	39 (20.6)	
	Overweight (26–30)	25 (10.0)	19 (76.0)	6 (24.0)	
	Obese (> 30)	3 (1.2)	3 (100.0)	0 (0.0)	
**Consumption of restaurant food**	Always	3 (1.2)	3 (100.0)	0 (0.0)	0.840
	Often	84 (33.7)	66 (78.6)	18 (21.4)	
	Sometimes	153 (61.4)	122 (79.7)	31 (20.3)	
	Never	9 (3.6)	7 (77.8)	2 (22.2)	
**Tea/coffee consumption**	Never	70 (28.1)	60 (85.7)	10 (14.3)	0.458
	1–3 cups/day	169 (67.9)	131 (77.5)	38 (22.5)	
	> 3 cups/day	10 (4.0)	7 (70.0)	3 (30.0)	
**Skipping breakfast**	Never skip	83 (33.3)	66 (79.5)	17 (20.5)	0.519
	Skip sometimes	64 (25.7)	54 (84.4)	10 (15.6)	
	Often skip	65 (26.1)	48 (73.8)	17 (26.2)	
	Skip every day	37 (14.9)	30 (81.1)	7 (18.9)	
**Physical exercise**	Yes	111 (44.6)	93 (83.8)	18 (16.2)	0.135
	No	138 (55.4)	105 (76.1)	33 (23.9)	
**Frequency of exercise**	No exercise	86 (34.5)	67 (77.9)	19 (22.1)	0.745
	< 5 times/week	89 (35.7)	69 (77.5)	20 (22.5)	
	≥ 5 times/week	47 (18.9)	39 (83.0)	8 (17.0)	
	Every day	27 (10.8)	23 (85.2)	4 (14.8)	
**Mode of exercise**	No exercise	75 (30.1)	59 (78.7)	16 (21.3)	0.034*
	Walking briskly	128 (51.4)	108 (84.4)	20 (15.6)	
	Gym	27 (10.8)	16 (59.3)	11 (40.7)	
	Jogging	19 (7.6)	15 (78.9)	4 (21.1)	
**Duration of exercise**	No exercise	83 (33.3)	66 (79.5)	17 (20.5)	0.995
	< 30 min	71 (28.5)	56 (78.9)	15 (21.1)	
	30 min	37 (14.9)	30 (81.1)	7 (18.9)	
	> 30 min	58 (23.3)	46 (79.3)	12 (20.7)	
**Aerated drink consumption**	Always	5 (2.0)	4 (80.0)	1 (20.0)	0.248
	Often	55 (22.1)	41 (74.5)	14 (25.5)	
	Sometimes	143 (57.4)	117 (81.8)	26 (18.2)	
	Never	46 (18.5)	36 (78.3)	10 (21.7)	
**Midnight snacking**	Always	8 (3.2)	3 (37.5)	5 (62.5)	0.007*
	Often	56 (22.5)	43 (76.8)	13 (23.2)	
	Sometimes	123 (49.4)	97 (78.9)	26 (21.1)	
	Never	62 (24.9)	55 (88.7)	7 (11.3)	
**Tobacco/nicotine use**	Daily	1 (0.4)	1 (100.0)	0 (0.0)	0.717
	Frequently	2 (0.8)	1 (50.0)	1 (50.0)	
	Occasionally	9 (3.6)	7 (77.8)	2 (22.2)	
	Never	237 (95.2)	189 (79.7)	48 (20.3)	
**Smoking status**	Yes	2 (0.8)	1 (50.0)	1 (50.0)	0.299
	No	247 (99.2)	197 (79.8)	50 (20.2)	
**Past medical illness**	Yes	55 (22.1)	41 (74.5)	14 (25.5)	0.301
	No	194 (77.9)	157 (80.9)	37 (19.1)	
**Perceived stress level (PSS)**	Low Stress	40 (16.1)	32 (80.0)	8 (20.0)	0.025*
	Moderate Stress	178 (71.5)	147 (82.6)	31 (17.4)	
	High Stress	31 (12.4)	19 (61.3)	12 (38.7)	

* Statistically significant (*p* < 0.05).

The prevalence of GERD was found to be 20.5% (n = 51) among the respondents based on self‐reported symptoms (participants scoring ≥ 8 on the GERDQ) as shown in Figure 1.

**Figure 1:**
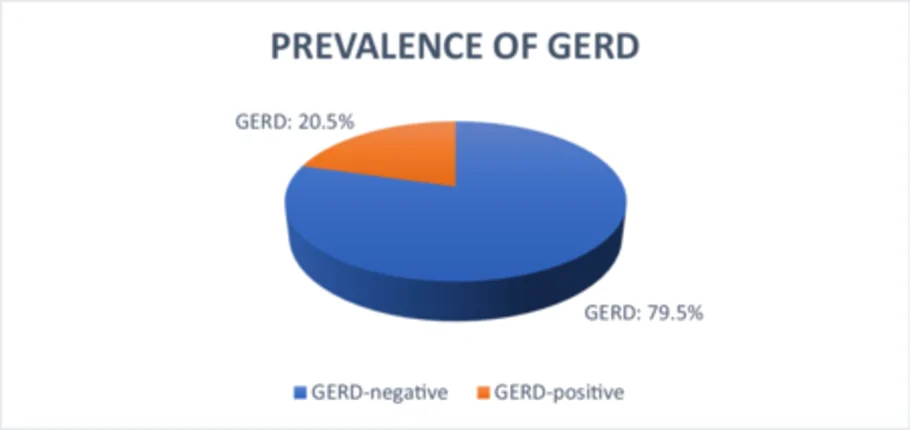
Distribution of GERD status among study participants.

While there was no significant relationship between the prevalence of GERD and sex, BMI, and academic year, among lifestyle risk factors, we were able to observe a statistically significant association (*p*‐value < 0.05) between the GERD status and midnight snacking, mode of exercise and perceived stress (*p* < 0.05).

Additionally, 35.3% and 40.2% of the participants complained of experiencing heartburn and regurgitation at least once a week, respectively. Nausea was the most prevalent symptom experienced at least once weekly by 48.6% of the participants, as shown in Table 2.

**Table 2:** Frequency of GERDQ symptoms among participants.

	n (%)			
	0 days	1 day	2 or 3 days	4 or 7 days
Burning feeling behind the breastbone/sternum	161 (64.7)	56 (22.5)	24 (9.6)	8 (3.2)
Stomach contents moving up to the throat or mouth (regurgitation)	149 (59.8)	66 (26.5)	26 (10.4)	8 (3.2)
Pain in the middle of the upper stomach area (epigastric region)	150 (60.2)	62 (24.9)	32 (12.9)	5 (2.0)
How often do you have nausea	128 (51.4)	79 (31.7)	33 (13.3)	9 (3.6)
Trouble getting a good night's sleep because of heartburn or regurgitation	191 (76.7)	41 (16.5)	13 (5.2)	4 (1.6)
Need for over‐the‐counter medicine for heartburn or regurgitation (such as antacids), in addition to the medicine your doctor prescribed?	205 (82.3)	28 (11.2)	10 (4.0)	6 (2.4)

GERDQ and PSS scores were not normally distributed (*p* < 0.05), assessed using Shapiro‐Wilk test. Spearman's rank correlation revealed no significant correlation between observed GERDQ and PSS score (ρ = −0.022, *p* = 0.727, n = 249).

Variables with *p* < 0.20 in the bivariate analysis were analyzed in the multivariate logistic regression model. The model was statistically significant (χ^2^ (8) = 24.636, *p* = 0.002), accounting for 14.8% of the variance in GERD. Participants with a habit of midnight snacking showed 7.6 times higher odds of probable GERD compared to consuming them sometimes (OR 7.62, 95% CI 1.60–36.21). Similarly, participants doing heavy lifting/gym‐based exercise had higher odds of GERD compared to those who walked briskly (OR = 4.00, 95% CI: 1.53–10.46, *p* = 0.005). After adjusting for the other variables, the model revealed that stress was no longer the predictor of probable GERD, as seen in Table 3.

**Table 3:** Multivariate regression analysis of factors associated with GERD.

Predictor	Category	B	SE	AOR (Exp(B))	95% CI	*p* value
Midnight Snacking	Always	2.03	0.80	7.62	1.60–36.21	0.011
	Often	0.30	0.41	1.35	0.61–3.00	0.465
	Never	−0.58	0.47	0.56	0.22–1.41	0.215
Mode of Exercise	No exercise	0.39	0.39	1.48	0.69–3.19	0.316
	Jogging	0.41	0.65	1.51	0.42–5.41	0.532
	Gym	1.39	0.49	4.00	1.53–10.46	0.005
Perceived Stress	Moderate	−0.06	0.47	0.94	0.37–2.37	0.894
	High	1.06	0.58	2.89	0.92–9.09	0.069

*Note:* Model fit: χ^2^ (8) = 24.636, *p* = 0.002; Nagelkerke R^2^ = 0.148.

## 4 Discussion

This is one of the first nation‐based studies to investigate the probable prevalence of GERD among medical students in Pakistan. The goal was to identify the important and reversible predictors that lead to the development of this disease, such as lifestyle choices, and outline various risk factors that require further investigation. Our study included a relatively diverse sample of 249 participants, the majority of whom were female, in their second year of academic study, and had a healthy BMI.

GERD affected approximately one‐fifth of medical students, or 20.5%, a significant proportion compared to the general public's 16.0%, as reported in 2021 [20]. Researchers in southern India found that 30% of undergraduate medical students typically experience at least one instance of heartburn or regurgitation within a week [21]. In line with previous studies, GERDQ has been used for assessment and has been reported to have relatively high specificity and sensitivity. However, this assessment method includes self‐reported GERD rather than a clinically confirmed diagnosis; it should be interpreted cautiously, as it may influence the probable prevalence. Furthermore, the present literature has consistently shown relatively higher prevalence rates of GERD [12]. The most plausible cause could be that clinical settings tend to capture symptomatic and severely affected individuals and are corroborated by diagnostic testing, while our research may reflect symptom reporting behavior rather than true disease burden. Moreover, underreporting the symptoms due to normalization of discomfort or self‐management of symptoms like self‐medication can also be the cause. The sample comprises a more specific and homogeneous group of the younger population, which may explain the absence of more severe manifestations of GERD, as advancing age and prolonged exposure to risk factors tend to contribute to its severity.

Unlike the 2022 Mendelian randomization study, there was no significant association between GERD and smoking in our study. This can mainly be attributed to the relatively small number of smokers within our sample, thereby reducing statistical power to detect any meaningful effect [12]. Furthermore, there was no significant relationship between the prevalence of GERD and age, academic year, or sex in our study; however, inconsistent findings are present in the existing literature regarding sex and GERD prevalence [22, 23]. The sex imbalance in the sample and selection bias may have limited the ability to identify sex‐based differences, and therefore, results should be considered context‐specific. This study also found no significant link between obesity and GERD. This is in contrast to prior research, which suggested a positive relationship between BMI and GERD symptoms [24, 25]. Methodological limitations, such as self‐reported anthropometric data, limited variability, the younger age profile of the sample, and the absence of the compounding effect of obesity in association with reflex disease, may explain this variation.

In East Asian populations, analyses have suggested that tea consumption might increase the risk of GERD; however, this study has failed to establish a conclusive link between tea consumption and GERD [26]. This variation can be attributed to the nature of self‐reported data, which introduces recall bias and exposure misclassification, the younger age of our sample, and individual tolerance to caffeinated beverages, causing discrepancies in the assessed outcome.

Our investigation revealed a significant association between midnight snacking, mode of exercise and perceived stress with the prevalence of GERD. Due to the limited explanatory power of the model, GERD occurrence cannot be solely explained by these lifestyle factors. Current literature is divided regarding the association between poor dietary habits and GERD [27, 28]. However, the majority of the literature shows a significant correlation between GERD and a short interval between dinner and sleep, specifically less than 2‐3 h, explained by the physiological delay in gastric transit during rest, further corroborating our findings [29]. However, details of portion size, precise meal timings and dietary composition could have provided more in‐depth details of the exposure.

Additionally, the link between physical activity (PA) levels and GERD prevalence explored was found to be similar to that reported in previous studies [22]. Physical activity encompasses diverse exercises and intensities, influencing disease prevention and rehabilitation [30]. In the multivariate analysis, it was revealed that heavy lifting or gym‐based exercise contributed to GERD compared to brisk walking. The existing literature reinforces this finding by explaining how vigorous activities leading to raised intra‐abdominal pressure compromise LES function and promote the reflux of gastric contents [31, 32]. Nonetheless, the cross‐sectional design of this study cannot eliminate reverse causation and should be considered when evaluating these results.

Similarly, 71.5% of medical students experienced moderate levels of perceived stress, whereas 12.4% experienced high perceived stress. Although perceived stress was significantly associated with GERD in the bivariate analysis, the relationship was no longer evident in the multivariate model. Chronic stress is a major contributor to the exacerbation of GERD symptoms, with research revealing a strong correlation between stress and reflux esophagitis. Medical students are disproportionately affected by the relentless pressure from academic demands. Stress triggers a trio of adverse effects, such as heightened gastric acid production, slowed gastric emptying, and delayed reflux, ultimately leading to worsened GERD symptoms [33, 34]. The discrepancy in our findings can be explained by the use of self‐reported stress measures, which introduced reporting bias to the data, and the adjustment of other factors led to the attenuation of this association. Furthermore, lifestyle factors such as eating habits and exercise may have partially confounded the observed association.

This study revealed several controllable risk factors of GERD. In Pakistan, the growing number of these predictors underscores the need for preventive measures. Future longitudinal studies are required to further investigate the specific relation of GERD and its risk factors and to determine effective interventions to address this growing burden.

### 4.1 Limitations

This study had several important limitations that should be considered when interpreting the findings. The cross‐sectional nature of this study precludes causal inference, emphasizing the need for longitudinal research to elucidate the directionality and causality of observed relationships. The absence of a pilot study may have impacted the calibration of the adapted questionnaire, and the use of self‐reported data, especially for GERD symptoms and lifestyle factors, may have introduced recall bias, potentially affecting the validity and reliability of the results. Furthermore, due to the non‐probability nature of the sampling technique and online recruitment, the findings from this study have limited representativeness and generalizability to all medical students in Pakistan, even though efforts were made to reach a geographically diverse sample.

The absence of formal diagnostic confirmation through medical examinations or endoscopy may contribute to misclassification of GERD status; however, GERDQ remains a practical tool for epidemiological studies to estimate symptom‐based prevalence and has been validated in several populations. This study did not extensively explore the cultural and regional factors influencing lifestyle behaviors, and social desirability bias, e.g., smoking, which further affects the generalizability of the findings. Finally, the study sample was predominantly female (85.1%), which may reflect the actual distribution in medical colleges of Pakistan; however, this can also be the result of selection bias and may have potentially influenced the assessment of exposure and outcome. Nonetheless, this study provides useful preliminary insights, and addressing these limitations in future research efforts by gender‐balanced and stratified sampling will contribute to a more comprehensive understanding of GERD among university students in Pakistan and beyond.

## 5 Conclusion

GERD affected one in five medical students and was associated with perceived stress, physical inactivity, and midnight snacking. These findings encourage the potential need for the incorporation of stress management programs and support groups, fostering healthier lifestyle behaviors, disease‐specific awareness, and warranting further evaluation through future prospective and interventional studies.

## Author Contributions

**Easha Masood:** conceptualization, methodology. **Sana Fatima:** software. **Rumman Ashraf:** data curation. **Muddassir Khalid:** project administration, writing – review and editing. **Fatima Tul‐Zahra:** investigation, data curation, visualization. **Mishaal Zarar:** validation, formal analysis, writing – original draft. **Sumia Fatima:** supervision, writing – review and editing. **Fred Segawa:** project administration, writing – review and editing. All authors have read and approved the final version of the manuscript. Fred Segawa had full access to all of the data in this study and takes complete responsibility for the integrity of the data and the accuracy of the data analysis.

## Funding

The authors have nothing to report.

## Ethics Statement

Approval was given by the Ethical Review Board (ERB) of Rawalpindi Medical University. Informed consent was taken from every participant. In this study, all methods were carried out in accordance with relevant guidelines and regulations. The study adhered to the Declaration of Helsinki in this regard. The current research was done after obtaining the approval of the Ethics Committee of Rawalpindi Medical University, Rawalpindi, Pakistan (Ethical code: PH‐46‐45) on November 5, 2024.Anonymity and confidentiality were maintained at all stages.Written informed consent from the subjects was obtained.

## Consent

Written informed consent from the subjects was obtained.

## Conflicts of Interest

The authors declare no conflicts of interest, financial or otherwise, that could have influenced the research, affirming that this study was conducted independently and without any commercial or financial relationships that could be perceived as a potential conflict of interest.

## Transparency Statement

The corresponding author affirms that this manuscript is an honest, accurate, and transparent account of the study being reported; that no important aspects of the study have been omitted; and that any discrepancies from the study as originally planned have been explained.

## Data Availability

The data that support the findings of this study are available from the corresponding author upon reasonable request.
